# Hospital bed occupancy for rotavirus and all cause acute gastroenteritis in two Finnish hospitals before and after the implementation of the national rotavirus vaccination program with RotaTeq®

**DOI:** 10.1186/s12913-014-0632-z

**Published:** 2014-12-11

**Authors:** Susanne Hartwig, Matti Uhari, Marjo Renko, Perrine Bertet, Maria Hemming, Timo Vesikari

**Affiliations:** Epidemiology Department, Sanofi Pasteur MSD, Lyon, France; Department of Paediatrics, University of Oulu, Oulu, Finland; Vaccine Research Centre, University of Tampere Medical School, Tampere, Finland

## Abstract

**Background:**

Vaccination-impact studies of the live-attenuated pentavalent oral vaccine Rotateq® have demonstrated that the burden of rotavirus gastroenteritis has been reduced significantly after the introduction of RotaTeq® vaccination, but less is known about the benefit of this vaccination on hospital overcrowding.

**Methods:**

As part of an observational surveillance conducted during the RV seasons 2000/2001 to 2011/2012, we analysed hospital discharge data collected retrospectively from two Finnish hospitals (Oulu and Tampere), concerning ICD 10 codes A00-09 (acute gastroenteritis, AGE) and A08.0 (rotaviral acute gastroenteritis RV AGE). We estimated the reduction in the number of beds occupied and analysed the bed occupancy rate, for RV AGE and all cause AGE, among 0–16 year-old children, before and after the implementation of the RV immunisation program.

**Results:**

The rate of bed days occupied for RV AGE was reduced by 86% (95% CI 66%-94%) in Tampere and 79% (95% CI 47%-92%) in Oulu after RV vaccination implementation. For all cause AGE, reduction was 50% (95% CI 29% to 65%) in Tampere and 70% (95% CI 58% to 79%) in Oulu. Results were similar among 0–2 year-old children. This effect was also observed on overcrowding in both hospitals, with a bed occupancy rate for all cause AGE >25% in only 1% of the time in Tampere and 9% in Oulu after the implementation of the immunisation program, compared to 13% and 48% in the pre-vaccination period respectively. After extrapolation to the whole country, the annual number of prevented hospitalizations for all cause AGE in the post-vaccination period in Finland was estimated at 1,646 and 2,303 admissions for 0–2 and 0–16 year-old children respectively.

**Conclusions:**

This study demonstrated that universal RV vaccination is associated with a clear decrease in the number of bed days and occupancy rates for RV AGE and all cause AGE. Positive consequences include increase in quality of care and a better healthcare management during winter epidemics.

## Background

In the pre-vaccination era rotavirus (RV) has been estimated to be responsible for two thirds of hospitalizations and emergency department consultations for acute gastroenteritis (AGE) in children [1] and it was estimated that RV infections lead to a yearly average of about 700,000 outpatient visits, more than 87,000 hospitalizations and 231 death in Europe [2].

**Figure 1:**
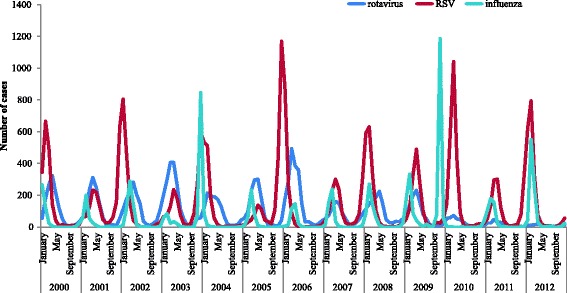
**Coincidence of rotavirus, RSV and influenza from national surveillance data in Finland.** From THL: http://www3.thl.fi/stat/.

Seasonal peaks of RV gastroenteritis occur yearly from November to May [3] and coincide in many countries with other winter epidemics such as bronchiolitis due to respiratory syncytial virus (RSV) and influenza [4-7] (Figure 1), leading potentially to overcrowding with increased risks for nosocomial infections and lessened quality of patient care. In the literature rotavirus has been repeatedly mentioned as a major cause of hospital overcrowding during the winter season [3,6,7].

The live-attenuated pentavalent oral vaccine Rotateq® was licensed in 2006. Postlicensure studies have shown high vaccine effectiveness in preventing hospitalization for RV gastroenteritis in the field, ranging from 83% to 98% [8-11], and consistent with pre-licensure efficacy results.

Furthermore, vaccination-impact studies have demonstrated that the burden of rotavirus gastroenteritis has been reduced significantly after the introduction of RotaTeq® vaccination. Evidence included reductions in healthcare utilization due to RV AGE (hospitalizations and emergency-department visits reduced by up to 90%), reductions in the magnitude and duration of the RV season as assessed by laboratory testing for RV, and the possible induction of herd immunity [12].

Less is known about the benefit of the implementation of immunization programs with Rotateq® to avoid situations of seasonal hospital overcrowding. Several studies have been conducted to evaluate overcrowding associated with AGE in emergency departments [13,14], with one study specifically assessing the workload in pediatric emergency units during the epidemic season [15], no study to our knowledge has measured seasonal hospital disruption due to RV gastroenteritis.

Several indicators have been studied to estimate emergency department crowding and two scales for quantifying crowding in emergency departments have been developed: NEDOCS and EDWIN [16-19]. However, no consensus and no indicators have been developed to measure hospital disruption. We propose to use bed occupancy rates as a proxy to assess situations of hospital overcrowding.

A prospective surveillance study was established in 2009 at two University hospitals in Finland (Tampere and Oulu) to document the number of cases of pediatric AGE requiring hospitalization, and the role of rotavirus and specific serotypes in those cases in the first three years after the implementation of the universal vaccination program with RotaTeq®. The study showed that after the implementation of universal RotaTeq® vaccination, RV AGE was virtually eliminated among children age-eligible for the vaccination program [11].

In the frame of this study, a retrospective analysis of the discharge database was performed to evaluate the impact of the introduction of RV vaccines into the national vaccination program on the rotavirus infections at hospitals.

The aim of the present analysis was to evaluate the benefit of the implementation of the National Immunization Program with Rotateq® to avoid situations of seasonal hospital overcrowding.

## Methods

The study was conducted in accordance with the Declaration of Helsinki, Good Pharmacoepidemiology Practice guidelines and local laws, rules and regulations. The ethics committee of Pirkanmaa Hospital District (Tampere) reviewed and approved the study protocol and amendments. The study was also approved by the head of each hospital.

In the frame of an observational surveillance study published elsewhere [11], hospital discharge data were retrospectively collected from Tampere and Oulu hospitals. We further analyzed the data of this retrospective study part for the RV seasons 2000/2001 to 2011/2012. The following information was extracted from the hospital information systems (discharge registers) and analyzed: ICD 10 codes A00-09 (all codes for intestinal infectious diseases), A08.0 (rotaviral enteritis).

Admission and discharge dates were used to calculate the number of beds occupied daily by infants and children diagnosed with RV AGE and all cause AGE (including RV AGE) during the RV seasons 2000/2001 to 2011/2012. In some cases, e.g. when patients were transferred from one hospital unit to another, or if a second admission occurred less than 14 days after a first discharge and if both admissions were coded with different ICD discharge codes (among A00-A09), one of them being A08.0 (the code for RV AGE), this one was selected as this code was only allocated in case of positive routine laboratory ELISA test result. In addition to clinical diagnostic, some samples are sometimes taken as part of the usual care of patients in both hospitals. In this case, PCR was done to identify precisely the type of RV causing the disease. Consequently, most reliable figures are the all cause AGE which do not depend on local diagnostic and testing practices.

The 0–16 year age group was chosen to follow hospital admissions including age groups not targeted by the vaccination, but who may be subject to herd immunity.

The period of 2000/01 to 2005/06 was considered as the pre-vaccination period, the period of 2006/07 to 2008/09 as a transition period during which both rotavirus vaccines (RotaTeq® and Rotarix®) were available in Finland, but with lower coverage rates (reaching 30-35%) [20], as the vaccines were not included into the national vaccination program. Universal routine vaccination against RV AGE was started in Finland on September 1st, 2009 and the period of 2009/10 to 2011/12 was thus considered as post-vaccination period. Each season started the 1^st^ of September and ended the 31^th^ of August of the following year.

The total number of bed days for RV AGE and for all cause AGE was reported by vaccination periods and seasons for each hospital. To assess the impact of the national RV vaccination program, two statistics were calculated. The first was the relative reduction of the annual number of bed days between the post- and pre-vaccination periods, and the second was the absolute reduction (difference) (95% CI) of the number of bed days, based on a vaccine probe approach [21]. For both estimates, we calculated 95% confidence interval (CI) based on the negative binomial distribution in order to take into account for over dispersion. Rates of bed days per 100,000 children were calculated based on children population figures from Statistics Finland available at http://www.stat.fi/til/vaerak/tau_en.html.

The proportions of RV positive cases among all causes AGE were also described per period and season and trend in these proportions between the post- and pre-vaccination periods was studied using a chi-squared test for trend in proportions.

We compared the number of beds occupied for all cause AGE and available beds per day, by estimating a bed occupancy rate defined as the ratio between the number of beds occupied for all cause AGE and the number of available beds in Tampere (n = 16) and in Oulu (n = 9). The bed occupancy rate was then stratified in 5 classes, 0-25%, 26-50%, 51-75%, 76-100% and >100%. The distribution of days according to the defined bed occupancy classes was compared between the post- and pre-vaccination periods using a chi-squared test.

We extrapolated the results of this study to the whole country by estimating the 2013 annual absolute number of bed days and prevented bed days after the introduction of the national RV program in Finland. For this, we pooled the data of Tampere and Oulu by period (i.e. pre- and post-vaccination) to calculate the rates (and its 95% CI) of number of bed days and number of prevented bed days per 100,000 children aged respectively 0–16 and 0–2. Then, we applied these rates to the whole national population figures of children 0–16 and 0–2 years in 2013. Population figures were extracted from Statistics Finland, available at: http://www.stat.fi/til/vaerak/tau_en.html. Analyses were done using SAS, SPSS and R, and *p*-values <0.05 were considered as statistically significant.

## Results

In Tampere, the overall number of bed days per 100,000 0–2 year-old children for RV AGE decreased from 925 in the pre-vaccination to 70 in the post-vaccination period corresponding to a statistically significant reduction of 93% (95% CI 77%-98%) (Table 1). Similar results were observed when enlarging the age group to children up to 16 years, with the overall number of bed days per 100,000 children for RV AGE decreasing from 220 to 31, corresponding to a reduction of 86% (95% CI 66%-94%). For all cause AGE, a lower decrease from 2,839 bed days per 100,000 children in the pre-vaccination era to 972 bed days per 100,000 children in the post-vaccination era was observed among 0-2y children, corresponding to a reduction of 66% (95% CI 49% to 77%) and from 886 to 440 bed days per 100,000 children, thus a 50% reduction (95% CI 29% to 65%) among 0-16y children. Table 1 also presented the corresponding absolute reduction of the number of bed days per 100,000 children.

**Table 1:** **Number of bed days, reduction of the number of bed days for RV and all cause AGE, and proportion of RV among all cause AGE according to vaccination period and season among 0-16y and 0-2y children in Tampere**

		**Children 0–16 years**				**Children 0–2 years**			
**Period**	**Years**	**Number of children in the catchment area**	Number of bed days*		**% RV among all AGE**	**Number of children in the catchment area**	Number of bed days*		**% RV among all AGE**
			**RV AGE**	**All AGE**			**RV AGE**	**All AGE**	
**Pre-vaccination period**	2001-2002	87,856	238	839	28.4	15,241	181	477	37.9
	2002-2003	88,105	288	1098	26.2	15,361	185	563	32.9
	2003-2004	88,112	197	561	35.1	15,719	174	379	45.9
	2004-2005	88,373	165	614	26.9	16,237	140	375	37.3
	2005-2006	88,485	80	793	10.1	16,698	53	456	11.6
	Annual average	**-**	193.6	781.0	24.8	**-**	146.6	450	32.6
	Overall number of bed days per 100,000 children	**-**	220	886		**-**	925	2839	
**Transition period**	2006-2007	88,657	43	600	7.2	16,932	29	329	8.8
	2007-2008	88,591	290	1010	28.7	17,201	189	574	32.9
	2008-2009	88,799	191	688	27.8	17,502	128	345	37.1
	Annual average	**-**	174.7	766	22.8	**-**	115.3	416.0	27.7
	Overall number of bed days per 100,000 children	**-**	197	864		**-**	670	2417	
**Post-vaccination period**	2009-2010	88,919	18	417	4.3	17,655	9	163	5.5
	2010-2011	89,322	60	495	12.1	17,637	28	257	10.9
	2011-2012	90,046	6	269	2.2	17,798	0	96	0.0
	Annual average	**-**	28.0	393.7	7.1	**-**	12.3	172.0	7.2
	Overall number of bed days per 100,000 children	**-**	31	440		**-**	70	972	
**% reduction (95% CI) of the number of bed days per 100,000 children between the post- and pre-vaccination periods**			−86 (−66;-94)	−50 (−29;-65)			−93 (−77;-98)	−66 (−49;-77)	
**Absolute reduction (95% CI) of the number of bed days per 100,000 children between the post- and pre-vaccination periods**			−188 (−173;-204)	−445 (−408;-483)			−855 (−785;-926)	−1867 (−1723;-2011)	

*unless otherwise specified.

In Oulu, the average annual number of bed days for RV AGE per 100,000 0–2 year -old children decreased from 1,017 in the pre-vaccination to 170 in the post-vaccination period corresponding to a statistically significant reduction of 83% (95% CI 35%-96%) (Table 2). For 0–16 year-old children, the average annual number of bed days per 100,000 children for RV AGE decreased from 261 to 54, corresponding to a reduction of 79% (95% CI 47%-92%). For all cause AGE, a lower decrease from 3,850 bed days per 100,000 children in the pre-vaccination era to 757 bed days per 100,000 children in the post-vaccination era was observed among 0-2y children, corresponding to a reduction of 80% (95% CI 68% to 88%) and from 1,233 to 365 bed days per 100,000 children, thus a 70% reduction (95% CI 58% to 79%) among 0-16y children. Table 2 also presented the corresponding absolute reduction of the number of bed days per 100,000 children.

**Table 2:** **Number of bed days, reduction of the number of bed days for RV and all cause AGE, and proportion of RV among all cause AGE according to vaccination period and season among 0-16y and 0-2y children in Oulu**

		**Children 0–16 years**				**Children 0–2 years**			
**Period**	**Years**	**Number of children in the catchment area**	Number of bed days*		**% RV among all AGE**	**Number of children in the catchment area**	Number of bed days*		**% RV among all AGE**
			**RV AGE**	**All AGE**			**RV AGE**	**All AGE**	
**Pre-vaccination period**	2001-2002	83,846	207	861	24.0	15,161	142	483	29.4
	2002-2003	83,668	260	1127	23.1	15,310	169	655	25.8
	2003-2004	84,107	260	1056	24.6	15,818	216	633	34.1
	2004-2005	84,443	151	733	20.6	16,274	109	429	25.4
	2005-2006	84,870	219	1415	15.5	16,606	169	848	19.9
	Annual average	**-**	219.4	1038.4	21.1	**-**	161	609.6	26.4
	Overall number of bed days per 100,000 children	**-**	261	1233		**-**	1017	3850	
**Transition period**	2006-2007	85,064	33	1241	2.7	16,903	25	671	3.7
	2007-2008	85,257	84	840	10.0	16,906	31	387	8.0
	2008-2009	85,683	161	787	20.5	17,145	115	425	27.1
	Annual average	**-**	92.7	956.0	2.7	**-**	57	494.3	11.5
	Overall number of bed days per 100,000 children	**-**	109	1120		**-**	336	2910	
**Post-vaccination period**	2009-2010	86,144	116	433	26.8	17,312	84	221	38.0
	2010-2011	86,498	13.0	222	5.9	17,315	2	70	2.9
	2011-2012	87,264	11	294	3.7	17,285	2	102	2.0
	Annual average	**-**	46.7	316.3	14.8	**-**	29.3	131.0	22.4
	Overall number of bed days per 100,000 children	**-**	54	365		**-**	170	757	
**% reduction (95% CI) of the number of bed days per 100,000 children between the post- and pre-vaccination periods**			−79 (−47;-92)	−70 (−58;-79)			−83 (−35;-96)	−80 (−68;-88)	
**Absolute reduction (95% CI) of the number of bed days per 100,000 children between the post- and pre-vaccination periods**			−207 (−189;-225)	−868 (−828;-909)			−847 (−769;-926)	−3092 (−2937;-3249)	

*unless otherwise specified.

The proportion of RV positive cases among all cause AGE was also significantly reduced in the post-vaccination period compared to the pre-vaccination period in 0-16y children and 0-2y children from Tampere (Table 1) and in 0-16y children from Oulu (Table 2). The proportion of RV positive cases among all cause AGE in 0-2y children from Oulu did not significantly vary between vaccination periods (22.4% vs 26.4%, p = 0.087) and this was mainly due to the high proportion observed during the 2009/10 season.

**Figure 2:**
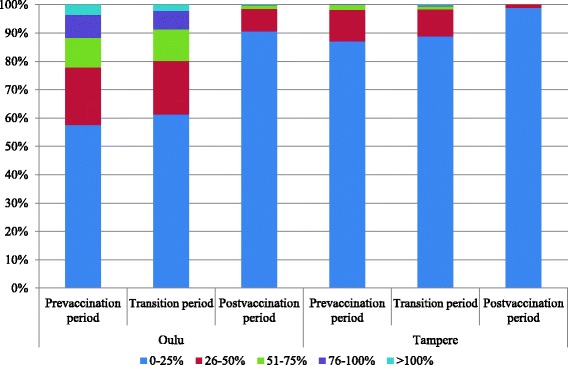
**Distribution of the number of days with bed occupancy of 0-25%, 26-50%, 51-75%, 76-100% and >100% for all cause AGE in children 0–16 years in Oulu (9 beds) and Tampere (16 beds).**

In both hospitals in Oulu and in Tampere, bed occupancy for all cause AGE was significantly lower in the post-vaccination period than in the pre-vaccination period (Table 3, Figure 2). In Oulu, while bed occupancy for all cause AGE was >25% during 774 (48%) days in the pre-vaccination period, it decreased to 103 (9%) in the post-vaccination period. In Tampere, these figures were 235 (13%) in the pre-vaccination period and 12 (1%) in the post-vaccination period.

**Table 3:** **Distribution of the number of days with bed occupancy of 0-25%, 26-50%, 51-75%, 76-100% and >100% for all cause AGE in children 0–16 years in Oulu (9 beds) and Tampere (16 beds)**

**Season**	**Vaccination period**	**Total number of days**	**Bed occupancy for all cause AGE in children 0–16 years in Oulu**					**Bed occupancy for all cause AGE in children 0–16 years in Tampere**				
			**0-25%**	**26-50%**	**51-75%**	**76-100%**	**>100%**	**0-25%**	**26-50%**	**51-75%**	**76-100%**	**>100%**
2001-02	Pre-vaccination period	365	219	89	46	11	0	324	38	3	0	0
2002-03	Pre-vaccination period	365	196	79	41	37	12	273	75	17	0	0
2003-04	Pre-vaccination period	366	197	76	53	33	7	347	19	0	0	0
2004-05	Pre-vaccination period	365	247	78	21	18	1	337	26	2	0	0
2005-06	Pre-vaccination period	365	193	48	30	47	47	310	44	10	1	0
2006-07	Transition period	365	181	72	53	47	12	343	22	0	0	0
2007-08	Transition period	366	249	65	30	12	10	301	48	11	3	3
2008-09	Transition period	365	242	69	39	14	1	329	35	1	0	0
2009-10	Post-vaccination period	365	309	41	12	2	1	357	8	0	0	0
2010-11	Post-vaccination period	365	348	17	0	0	0	361	4	0	0	0
2011-12	Post-vaccination period	366	336	29	1	0	0	366	0	0	0	0
Total (%)	Pre-vaccination period	1826	1052 (58)	370 (20)	191 (10)	146 (8)	67 (4)	1591 (87)	202 (11)	32 (2)	1 (0)	0 (0)
	Transition period	1096	672 (61)	206 (19)	122 (11)	73 (7)	23 (2)	973 (89)	105 (10)	12 (1)	3 (0)	3 (0)
	Post-vaccination period	1096	993 (91)	87 (8)	13 (1)	2 (0)	1 (0)	1084 (99)	12 (1)	0 (0)	0 (0)	0 (0)

Table 4 shows the estimated national absolute number of bed days and number of prevented bed days after the introduction of the national RV immunization program in Finland which prevented 6,219 (95% CI, 5,956-6,483) bed days for all cause AGE for 0–16 year-old children and 4,443 (95% CI, 4,252-4,633) for 0–2 year-old children, leading to a number of bed days for all cause AGE of 3,846 (95% CI, 3,682-4,009) for 0–16 year-old children and of 1,552 (95% CI, 1,451-1,653) for 0–2 year-old children in 2013.

**Table 4:** **Estimation of the national number of bed days and the number of prevented bed days after the introduction of the national RV program in Finland. In 2013, national figures were 953,547 for children 0–16 years and 179,244 for children 0–2 years**

		**Rates per 100,000 children in 2012 based on Tampere and Oulu data**		**National estimates in 2013, after the introduction of the national RV program**	
		**Children 0–16 years**	**Children 0–2 years**	**Children 0–16 years**	**Children 0–2 years**
**Number of bed days (95% confidence interval)**	RV AGE	42 (37;48)	119 (98;140)	404 (352;458)	213 (176;251)
	All AGE	403 (386;420)	866 (809;922)	3,846 (3682;4009)	1,552 (1451;1653)
**Number of prevented bed days (95% confidence interval)**	RV AGE	197 (209;186)	852 (799;905)	1,880 (1769;1992)	1,527 (1432;1621)
	All AGE	652 (625;680)	2,479 (2372;2585)	6,219 (5956;6483)	4,443 (4252;4633)

The average length of stay for all cause AGE decreased significantly from 3.1 days in the pre-vaccination to 2.6 days in the post-vaccination period (p < 0.001) in Tampere and from 2.8 days to 2.4 (p < 0.001) in Oulu.

## Discussion

This study highlights the substantial impact of the national RV vaccination program on the reduction of the number of bed days for RV AGE and to a lesser extend for all cause AGE in two Finnish hospitals. Reduction in AGE hospitalizations non coded specifically as rotavirus suggest that testing and coding practices may underestimate the true rate of rotavirus hospitalizations as it has been described elsewhere [22]. Additionally, the mean length of hospital stay per episode decreased when comparing pre-vaccination periods with post-vaccination periods. Overall results suggest that universal vaccination with RotaTeq® led to a dramatic decrease of hospital overcrowding reducing the risk for nosocomial infections and situations of hospital disruption with negative adverse effects on the overall quality of patient care. These results are supported by the vaccine-probe design that allowed estimating the vaccine-preventable RV AGE and all AGE incidence, and extrapolate these results at the nationwide scale.

Our study presents some limitations. We cannot exclude that not all patients were tested, as hospitalization time may in some cases have been too short to allow children producing stool during their stay at hospital. When a laboratory result was not available at the time of hospital discharge of a patient, the case could not be recorded as RV AGE. Thus rotavirus as a cause for AGE is likely to be underestimated. We conclude that the reduction of the mean hospital length of stay could be attributable to the rotavirus vaccination program but cannot exclude that a modification of the hospital organization has occurred during the 10-year study period. Variability was observed in the population-based rates of severe rotavirus infections each year and this natural secular variability in rotavirus disease should be considered in the assessment of the impact of the vaccine, as it has been reported elsewhere [9,23,24]. This may explain the high rates of RV AGE in the transition period and the first year of the post vaccination period in the Oulu hospital.

In 2007 the Finnish National Institute for Health and Welfare (THL) estimated that without vaccination about 2,400 hospital admissions, 3,600 hospital outpatient visits, 9,000 primary care physician consultations and 0.5 deaths attributable to rotavirus would occur annually among children under 5 years of age in Finland [25]. When extrapolating the results of this study to the whole country, we estimated that 6,129 and 4,443 bed days for all cause AGE were prevented in 2013 by rotavirus vaccination in children 0–16 years and 0–2 years respectively. Assuming a hospital length of stay of 2.7 days (Oulu: 2.6 days and Tampere: 2.8 days) in the post-vaccination period, the annual number of prevented hospitalizations for all cause AGE can be estimated at 1,646 and 2,303 admissions for 0–2 and 0–16 year-old children respectively, thus confirming the assumptions made by Salo *et al*. in 2007 [25] who estimated that universal rotavirus vaccination could prevent some 2,000 hospital admissions in under five year-old children yearly.

Finland represents a privileged place to study RV disease and the impact of RV vaccination due to the high burden of RV and high vaccine uptake. It was one of the first countries in Europe recommending rotavirus vaccination in December 2007. In September 2009 it introduced universal rotavirus vaccination with exclusive use of the pentavalent human-bovine reassortant rotavirus vaccine RotaTeq® and following a vaccination schedule at age 2, 3 and 5 months reaching coverage rates of about 96% for the vaccines included in the NIP. In Finland, the RV related burden of disease was extensively studied through observational studies [26-28] and through data collected during the clinical development of rotavirus vaccines [2,29-32].

The impact of RV vaccination on the number of hospitalization was reported previously [11,32-36]. The present study describes that RV vaccination can also reduce seasonal hospital overcrowding due to RV AGE during winter epidemics by assessing the reduction in the number of occupied beds per day. While RV AGE hospitalization only describes the number of patients presenting at hospital with RV AGE, bed occupancy rather reflects the pressure that hospitals and health care workers face every day and specifically in periods of overlapping winter epidemics. Overcrowding occurs when new admissions for RV AGE exceed discharge of recovered patients, with adverse consequences such as lower quality of care [15], increased risk of nosocomial transmission among patients and healthcare workers (HCWs) [37,38], work stress [37-40], which may lead to hospital disruption with unsolved ethical and organizational issues [3,41-43].

We used bed occupancy to estimate crowding due to all cause AGE in children 0–16 year-old. While several indicators have been studied to estimate emergency department crowding with NEDOCS and EDWIN [16-19], no consensus and no indicators have been developed to measure hospital disruption. It may be reasonable to think that high occupancy rates (e.g. >50%) are associated with hospital disruption and impaired care. In addition to that a previous study conducted in a ward for paediatric infectious diseases at the Hospital of Oulu identified room sharing as one of the factors increasing the risk of hospital acquired infections [44].

## Conclusions

In conclusion, this study demonstrated that universal RV vaccination is associated with a clear decrease in the number of bed days and occupancy rates for RV AGE and all cause AGE. As a consequence, it is expected that RV vaccination increases quality of care by allowing a better management of overlapping winter epidemics, such as bronchiolitis or influenza and by decreasing associated costs for healthcare institutions.
